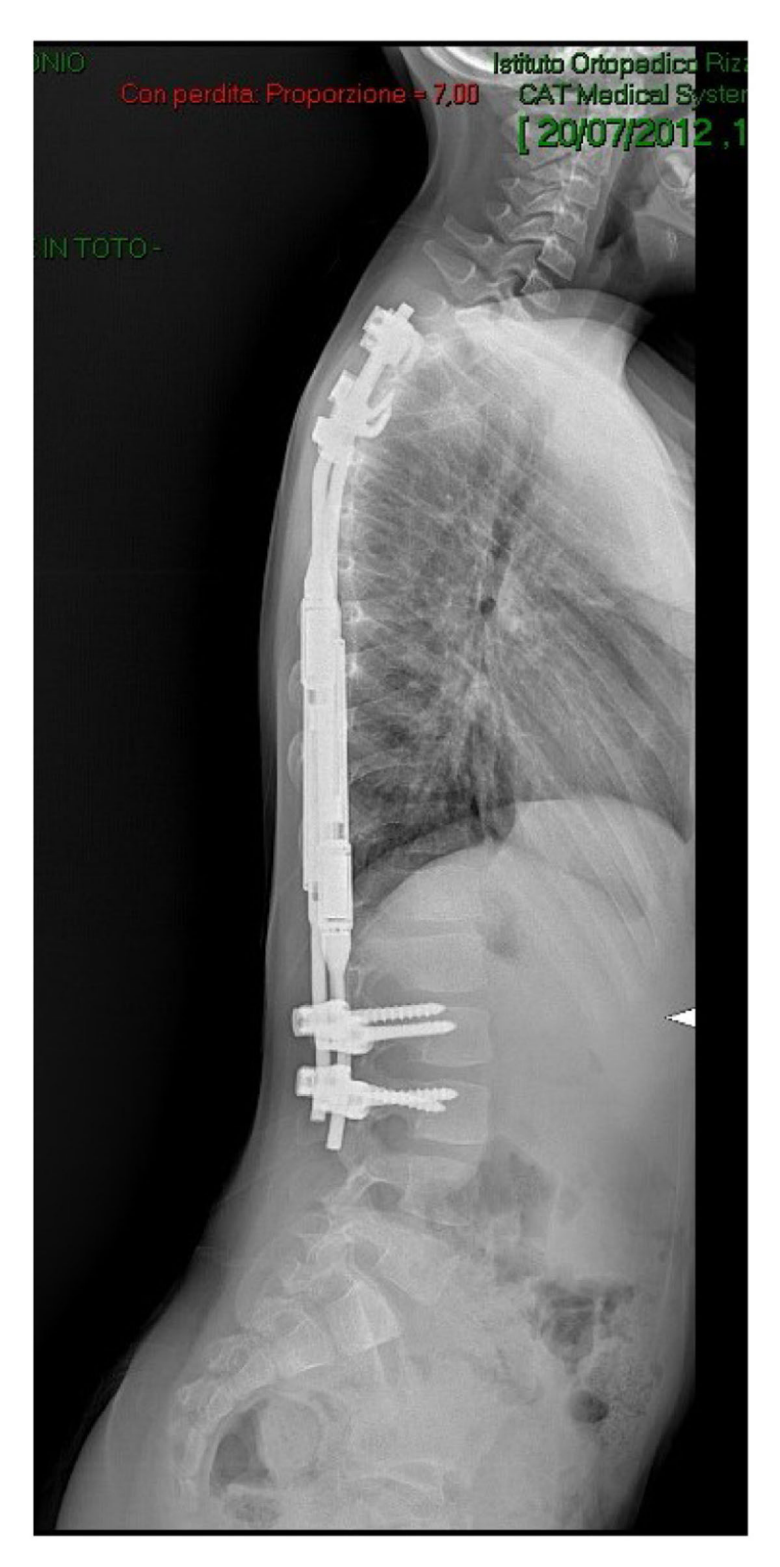# Magnetically controlled growing rod in early onset scoliosis

**DOI:** 10.1186/1748-7161-10-S1-O75

**Published:** 2015-01-19

**Authors:** Stefano Giacomini, Mario Di Silvestre, Francesco Lolli, Francesco Vommaro, Konstantinos Martikos, Elena Maredi, Andrea Baioni, Tiziana Greggi

**Affiliations:** 1 Deformities of Spine Surgery, Rizzoli Orthopaedic Institute, Bologna, Italy

## Background

Magnetically controlled growing rods (MCGR) are increasingly used for the treatment of early onset scoliosis. Aim of the study is to retrospectively review our patients treated with MCGR focusing on complications.

## Materials and methods

We retrospectively reviewed 7 patients, affected by early onset scoliosis and surgically treated with magnetically controlled growing rods (minimum follow up 6 months). There were 7 children, 3 females and 4 males, with an age ranging from 4 to 11 years. The aetiology was 6 idiopathic (infantile or juvenile), 1 congenital. In one case a VEPTR was first implanted before using MCGR.

In all cases a dual growing rod was implanted, using as distal anchors pedicle screws, as proximal anchors hooks.

## Results

At a minimum follow up of 6 months, after performing 5.7 lengthening procedures per patient (lengthening performed every 60-90 days), main thoracic scoliosis was corrected from 62.7° to 32.0° (mean correction 49%), lumbar curve form 58.5° to 32.0° (45%). The correction was maintained at final follow up. No neurological or infective complications occurred. In one patient a revision surgery was performed due to persistent sciatica secondary to lumbar misplaced screw. In another one, a revision was performed due to proximal hooks mobilization. At final follow up, no patient presents pain or functional limitation.

## Conclusions

Those results showed that MCGR can be safely and effectively used in patients affected by early onset idiopathic scoliosis, with an acceptable complications incidence (33%) if compared with literature regarding growing spinal implants, offering excellent deformity control and functional outcome.

**Figure 1:**
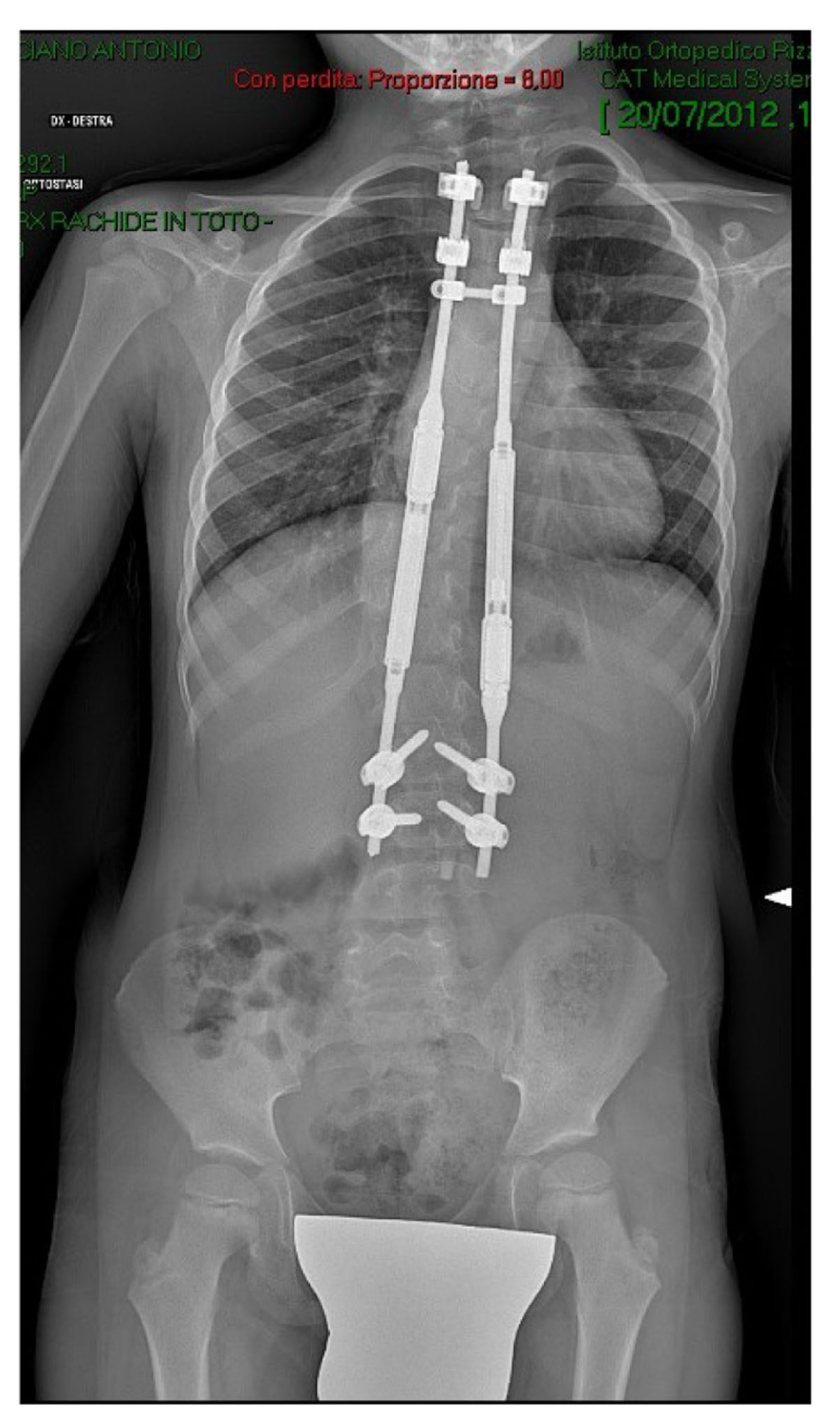


**Figure 2:**